# Thin Wall Milling at a Maximized Axial Depth of Cut: An Analysis of Thermal and Mechanical Interactions

**DOI:** 10.3390/ma18235347

**Published:** 2025-11-27

**Authors:** Magdalena Zawada-Michałowska

**Affiliations:** Department of Production Engineering, Lublin University of Technology, Nadbystrzycka 38D, 20-618 Lublin, Poland; m.michalowska@pollub.pl

**Keywords:** milling, thin-walled element, aluminium alloy, cutting force, cutting temperature, deformation, chatter, ANOVA, RSM, DoE

## Abstract

This paper reports the results of a study examining the effect of thermomechanical interactions that occur during a milling process conducted at a maximum axial depth of cut for a thin wall made of aluminium alloy 7050 T7451. The impact of cutting speed and wall thickness on cutting force and cutting temperature was determined. Response surface methodology and face-centred central composite design were used. It was found that raising the cutting speed to approximately *v_c_* ≈ 700 m/min led to an increase in cutting force component *F_x_* and cutting temperature *T*, followed by a decrease in their values. Nonetheless, these variables at *v_c_* = 900 m/min were considerably higher than those observed at *v_c_* = 300 m/min. The thinnest tested wall of *t* = 1 mm exhibited the greatest process instability and evident signs of chatter, while a wall thickness increase to *t* = 2 mm resulted in improved process stability and reduced flatness deviation. The interaction between the cutting force and the cutting temperature, as well as the occurrence of chatter, were established as two dominant factors affecting thin wall machining accuracy. Results showed that the assumed empirical models could be used to predict the tested dependent variables under similar milling conditions.

## 1. Introduction

In recent years, the aircraft industry has increasingly focused on finding solutions to reduce aircraft weight while at the same time increasing their reliability and operational efficiency [1,2]. Integral thin-walled components play a key role in this regard, as they are characterised by a relatively high strength [3] and are subject to narrow dimensional and geometric tolerances [4]. Their application contributes to a decrease in fuel consumption, which has a direct impact on reducing operating costs [5]. Thin-walled structural parts used in aircraft design include: stringers, ribs, frames, spars, shells, bulkheads, and skin panels [6,7]. Depending on their type and intended use, such elements are mainly made of light metal alloys, especially aluminium alloys [8], titanium alloys [9], and sometimes magnesium alloys [10]. Composite materials offer an alternative to these alloys [11], but their fabrication poses many problems [12]. Milling is the main method of machining thin-walled components for aircraft [13]. Given that these parts are designed to have a uniform structure [14] and rolled plates are currently applied as semi-finished products, with more than 90% of a plate becoming chips after machining [15,16]. It is reported that the buy-to-fly ratio (i.e., a ratio of the weight of the semi-finished product to the weight of the manufactured part) for such cases is up to 30:1 [17]. It helps avoid additional assembly operations and allows the production of a monolithic structure that once consisted of up to several hundred parts [18].

The machining of elements with thin walls requires a special technological approach. This is primarily related to the low rigidity of such components [19] and the resulting susceptibility to post-machining deformation [20] as well as chatter. These phenomena are the most commonly seen and troublesome problems [21,22]. Their occurrence makes it difficult to achieve both the appropriate dimensional and shape accuracy and the required surface quality [23]. As a result, companies are often forced to make corrections or scrap defective parts, which leads to increased manufacturing costs [24,25]. Despite these difficulties, the machining of thin-walled elements remains an economically viable solution, and the optimisation of process conditions is one of the key directions of research in this area [26]. It should be noted that the industry is intensively searching for new solutions that would simultaneously increase the efficiency of milling thin-walled components and minimise post-machining deformation. In this context, a variety of approaches are used, including the selection of a suitable machining strategy [27], tool path optimisation [28], selection of specified cutting parameters [29] or high-speed cutting parameters [30], and innovative methods of clamping the workpiece in the chuck [31]. Cutting tool manufacturers [32,33] have proposed specialised cutting tool designs, including end mills with varied blade pitch and variable helical angle for milling high and thin walls over their entire height (this process is also known as milling at a maximum axial depth of cut). This leads to more efficient tool use, shorter machining times, lower production costs, and higher process efficiency.

The development of advanced cutting tool designs and modern machining strategies has not eliminated the post-machining deformation of thin-walled components, which still poses a significant difficulty in industry. The main source of this problem is residual stress [34], which is why its root causes are studied all over the world. Residual stress can be classified into: initial residual stress (or bulk residual stress) and machining-induced residual stress [35]. Yiyang et al. [36] found that during the manufacture of a semi-finished product, external forces and temperature led to the formation of initial residual stress in the workpiece. Due to material removal during the machining process, the existing residual stress is released and redistributed, and the machining-induced residual stress is generated. It contributes to the creation of a new state of equilibrium and a new distribution of the residual stress. Kuczmaszewski et al. [37] emphasised that machining-induced residual stress is typically attributed mainly to the effects of the cutting force and the heat generated in the cutting zone. For this reason, two basic models of machining-induced residual stress formation are distinguished: a mechanical model related to the action of the cutting force and a thermal model which is related to the interaction of heat. However, it should be emphasised that these are simplified models, as the first one omits the influence of temperature, while the other one does not take into account the impact of cutting force [38]. In a real machining process, the workpiece undergoes deformation due to the impact of high loads, high strain rates, and friction between the tool and the machined surface. In addition to that, the machining-generated heat is of vital importance, since a non-uniform distribution of that heat leads to the occurrence of temperature gradients in the machined layer. This may trigger local phase transitions and microstructure modifications, affecting the state of stress in the workpiece [39,40]. Importantly, as for stress located in the surface layer, compressive stress is predominantly caused by the action of cutting force, whereas tensile stress results from temperature effects [41]. As mentioned previously, complex thermomechanical interactions occur during the machining process. The value and sign of residual stress in the material therefore depend on both factors, as well as on which one of them is dominant [38]. Ma et al. [42] claim that researchers predominantly present residual stress as a function of machining parameters, emphasising that residual stress should be considered primarily as a result of combined mechanical and thermal interactions. Table 1 shows a comparison of initial residual stress and machining-induced residual stress.

One of the main issues in the milling of thin-walled elements is chatter, which is a serious and undesirable phenomenon affecting the quality of manufactured parts [22]. Chatter occurs as a dynamic response of the workpiece–tool–chuck–machine tool system to the force acting on this system [47]. As thin-walled structures are characterised by low rigidity, even a slight disturbance in the cutting force can lead to a dynamic instability and a rapid increase in chatter amplitude. The effects of this phenomenon are particularly significant, as the resulting deformation of parts often makes it impossible to meet the required tolerances [48,49,50]. Chatter leads to reduced surface quality, the formation of characteristic marks known as chatter marks, as well as element damage. A prolonged impact of chatter can also cause faster tool wear and lower dimensional and shape accuracy of products [51,52].

The objective of this research is to evaluate the thermomechanical interaction occurring during thin wall milling at a maximum axial depth of cut. The study focuses on identifying and evaluating the impact of wall thickness and cutting speed on cutting force as well as cutting temperature. It is a continuation of the author’s previous research on thin wall milling at a maximized axial depth of cut [53], in which particular attention was devoted to analysing the influence of wall thickness and cutting speed on the post-machining deformation and residual stress formation. To clarify the physical foundations of the observed effects, it was necessary to concentration on the thermomechanical interactions occurring during machining. The problem of these interactions constitutes the main subject matter of this paper. These investigations are part of a broader research cycle aimed at developing a comprehensive understanding of the mechanisms that govern the quality of thin-walled components that were milled with a maximized axial depth of cut.

## 2. Materials and Methods

**Figure 1:**
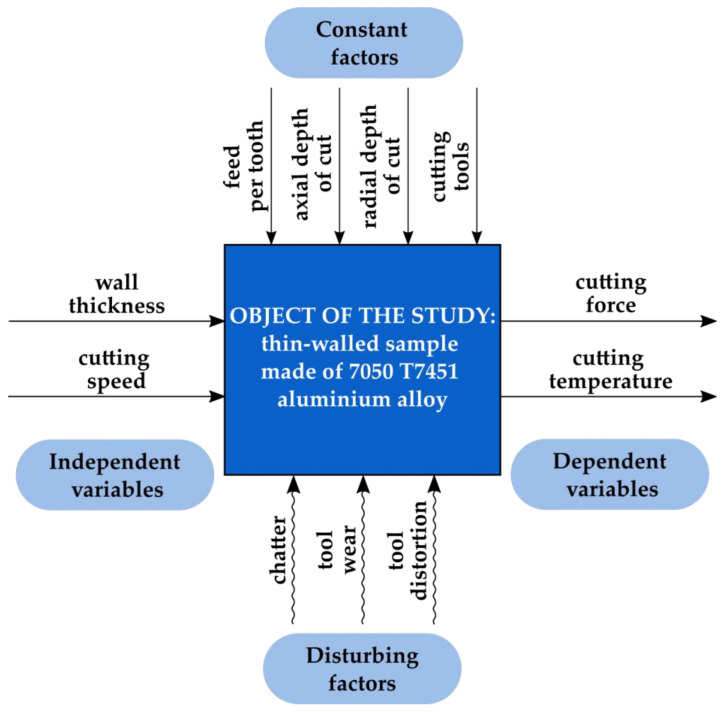
Design of the experiment.

Figure 1 shows the design of the experiment. A thin-walled sample with variable wall thickness, made of aluminium alloy 7050 T7451, was the object of the study. Experimental independent variables were thin wall thickness and cutting speed. Dependent variables related to thermomechanical interactions in the milling process were cutting force and cutting temperature. Constant factors included other technological parameters (feed per tooth, axial depth of cut, and radial depth of cut (milling width)), as well as cutting tools. Disturbing factors were chatter, tool wear, and tool deflection. 

**Figure 2:**
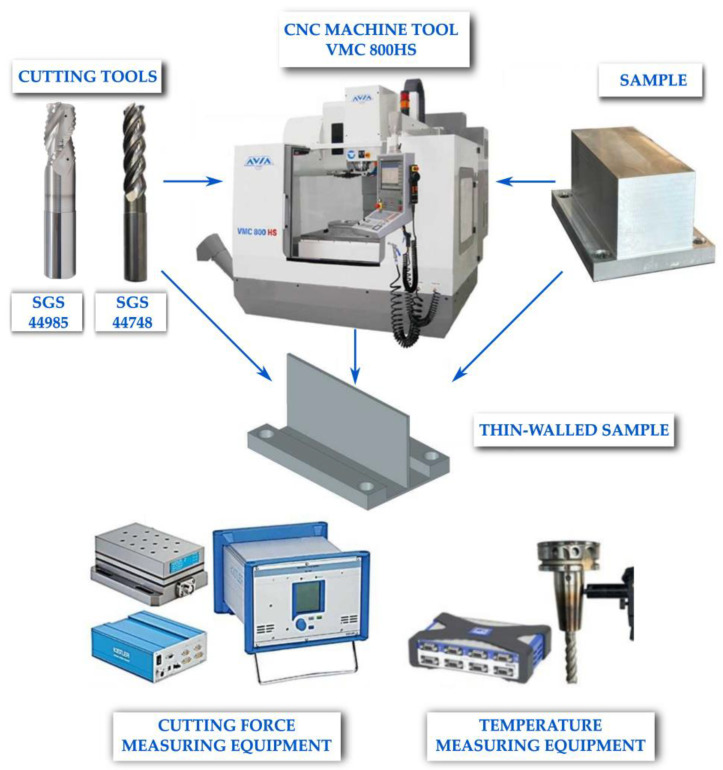
Experimental setup.

The experimental setup with all key components is shown in Figure 2. It included a CNC machine tool on which the cutting process was performed, as well as cutting tools selected according to the material properties and geometry of the workpiece. The figure also shows a sample before machining, as well as a milled part. The apparatus for examining thermal and mechanical interactions during the cutting process, including a dynamometer for measuring cutting force components and a stand for measuring cutting temperature using a natural thermocouple, was also presented. This approach allowed for a comprehensive investigation of both mechanical and thermal aspects of the cutting process, as well as a detailed interpretation of the obtained results.

The study was conducted in accordance with the design of experiments (DoE) procedure, which is an effective analytical tool for experimental design and analysis of experimental results, i.e., the development of a mathematical model that best fits the experimental data. Such an approach allows for the assessment of the impact of the independent variables on the dependent variables under study. It aims to obtain as much useful information as possible at minimal cost [54]. For this purpose, the specialist Design-Expert^®^ (23.1.8) software was applied, enabling comprehensive statistical analysis and visual processing of measurement data. The study employed response surface methodology (RSM), which is based on statistics and used for designing experiments and optimising process variables [55,56,57]. A face-centred central composite design (FCCCD) was selected to ensure a uniform distribution of experimental points in the experimental domain. Based on the applied design of experiment, requiring three variability levels for each test, the optimal number of indispensable experimental trials was set to 13. As required, the central point was replicated 5 times. Table 2 lists independent variables and their coded levels used in the face-centred central composite design. The variables were denoted by: *A*—wall thickness, and *B*—cutting speed. Each variable was coded at three levels: low (−1), medium (0), and high (+1).

The thin-walled samples were made of 7050 T7451 aluminium alloy, compliant with all the technical standards in the AMS4050K specification. This material exhibits very high strength and good resistance to corrosion cracking, particularly stress-corrosion cracking. The 7050 T7451 alloy is primarily used in aircraft applications for the production of fuselage, wing skin, frame, and other load-bearing structural components. The chemical composition and selected mechanical properties of this material are given in Table 3.

A rolled plate with a thickness of 50 mm was a semi-finished product. It was used to fabricate rectangular samples with the dimensions of 100 mm × 65 mm (length × width). The pretreatment involved preparing the workpiece for mounting in a specially designed mounting plate by means of screw joints, in order to ensure mounting repeatability and stability. Following the proper treatment, a thin wall with a height of 48 mm (which resulted from leaving a 2 mm thick base) and a variable thickness of 1 mm, 1.5 mm, and 2 mm was obtained.

**Figure 3:**
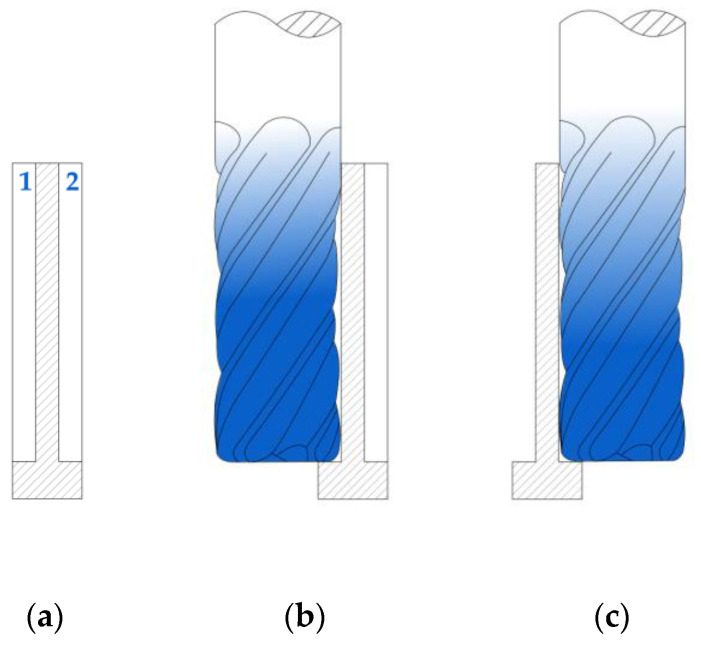
Finish milling of a thin wall at a maximized axial depth of cut: (**a**) wall before finishing, tool passes are denoted by 1 and 2; (**b**) first pass of the tool; (**c**) second pass of the tool.

Milling was conducted on the CNC Avia VMC 800HS machining centre. First of all, roughing was performed with constant technological parameters. The radial depth of cut (milling width) was adjusted in such a way that the finishing allowance value was the same, whatever the wall thickness. Finishing was carried out at a maximized axial depth of cut in two passes of the tool, one per side (Figure 3). It must be emphasised that both operations were performed as dry down-milling. Technological parameters of roughing and finishing operations are listed in Table 4.

The milling was conducted with the application of two cutting tools from KYOCERA SGS Precision dedicated to high-performance machining of aluminium alloys. Roughing was performed with a 44985 end mill, whereas finishing was performed with a 44748 end mill designed for thin wall machining at a maximum axial depth of cut. In both operations, ERICKSON AHPVTTMQL1C heat shrink tool holders 16090M (44985) and 12090M (44748) with an HSK63 tool taper were applied. They are manufactured by M & J Tooling LLC (M & J Tooling LLC, Dayton, OH, USA). It should be noted that the condition of the cutting edges was carefully inspected after the machining of each specimen using a Keyence VHX-5000 microscope (Keyence Corporation of America, Itasca, IL, USA). Detailed technical parameters of the end mill used for roughing and finishing are given in Table 5.

**Figure 4:**
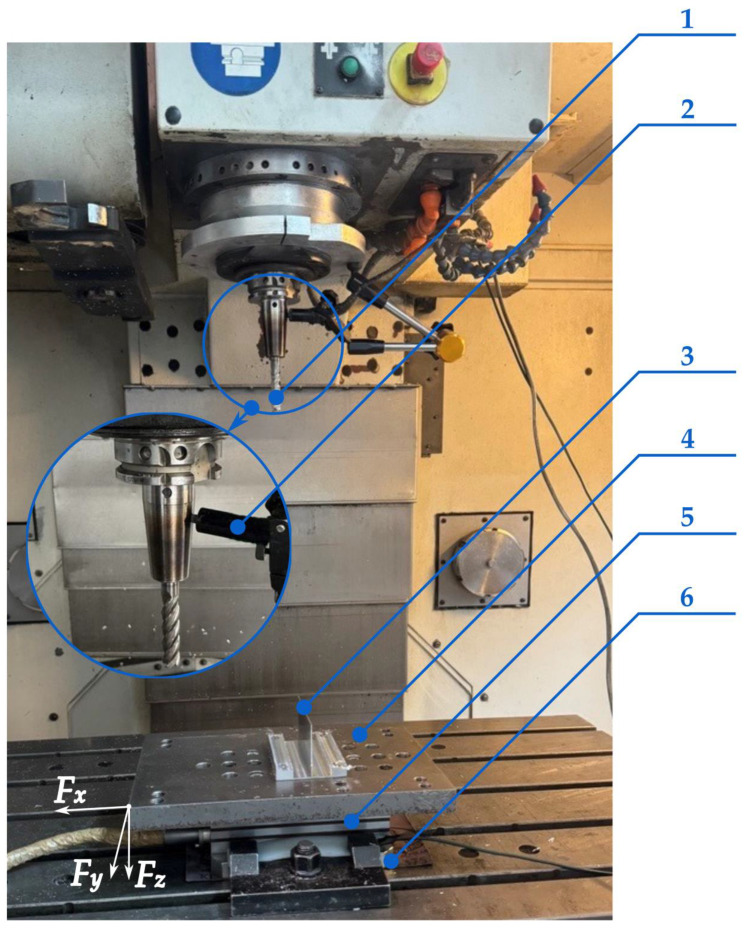
Stand for measuring cutting force components and cutting temperature (CNC machine tool workspace): 1—cutting tool (first junction of a natural thermocouple), 2—graphite brush, 3—workpiece (second junction of a natural thermocouple), 4—mounting plate, 5—dynamometer, 6—copper washer.

The study on the thermomechanical interactions occurring during thin wall milling at a maximized axial depth of cut focused on two dependent variables, i.e., the cutting force and the cutting temperature. Figure 4 shows a measuring stand that was located in the CNC machine tool workspace. Measurements of the cutting force components (*F_x_*, *F_y_*, *F_z_*) were made using a piezoelectric dynamometer Kistler 9257B (Kistler, Winterthur, Switzerland) that was mounted directly on the machine tool table. To its upper part, a mounting plate with a workpiece was fixed by screw joints. The measuring stand also comprised a signal amplifier Kistler 5070A (Kistler, Winterthur, Switzerland) and a data acquisition module DAQ 5697A (Kistler, Winterthur, Switzerland). Measurement data were processed in the DynoWare (type 2825A) software. The cutting temperature was measured on a specially designed measuring stand based on a natural thermocouple effect produced by the end mill and the workpiece. In this effect, a contact between two different materials—tool blade material and workpiece material—creates a thermocouple junction that generates an electromotive force proportional to the cutting temperature. According to the law of homogeneity for thermocouple junctions, the introduction of an additional conductive material into the thermocouple circuit does not affect the measurement, as long as both of its ends remain at the same temperature. Therefore, the use of supplementary components, i.e., the graphite brush, the copper washer, and the measurement cables transmitting the signal between the thermocouple junction and the amplifier, enabled signal acquisition without distortion of the results. Owing to the holder design with an integrated spring, the graphite brush remained pressed against the tool, ensuring a stable and disturbance-free electrical contact. The copper washer was placed beneath the dynamometer to isolate the system, which was necessary due to the complexity of the measurement setup. The obtained signal was transmitted to an amplifier HBM 1-MX840A (Hottinger Brüel & Kjær, Darmstadt, Germany) and recorded on a personal computer using the CatmanEasy (V35.1) software. The amplifier allowed for a high sampling frequency. For this case, 10,000 Hz was applied. Additionally, a dedicated stand was developed to calibrate the natural thermocouple formed by a sintered carbide (cutting tool material)—aluminium alloy (workpiece material) pair. The calibration data were used to establish a relationship between the electromotive force and the temperature. The calibration setup consisted of a natural thermocouple made of rods corresponding to the tool and workpiece materials. The thermocouple was placed inside a thermally insulated chamber equipped with heaters, and a reference thermocouple was attached at the junction of the two rods. The electromotive force generated by the natural thermocouple was recorded using the amplifier HBM 1-MX840A (Hottinger Brüel & Kjær, Darmstadt, Germany), while the temperature from the reference thermocouple was read with a multimeter MY-64 (XTREME, Stary Puznów, Poland). The calibration points were fitted with a fourth-degree polynomial, producing a coefficient of determination of *R*^2^ = 0.9970. The resulting relationship is presented in Equation (1):

(1) $$y=-4949.6x^{4}+9762.7x^{3}-6722.9x^{2}+2265.4x-48.661$$

where *y*—temperature in the cutting zone at the tool–workpiece interface [°C], *x*—electromotive force at the interface between the tool and workpiece materials [V].

Moreover, considering the author’s research presented in work [53], which related to thin wall milling at a maximized axial depth of cut and involved the analysis of the post-machining deformations, it was decided that the time curve of the cutting force component *F_x_* with the flatness deviation would be compared. These measurements were carried out using the Zeiss Contura 7/10/6 coordinate (Zeiss, Oberkochen, Germany) measuring machine, compatible with the Calypso (7.6.04) software and equipped with a VAST XT GOLD scanning probe (Zeiss, Oberkochen, Germany). A stylus with a 3 mm tip diameter was used. The obtained flatness deviation results are visualised in 3D form.

The methodology applied in this study made it possible to examine in detail the thermal and mechanical interactions occurring during thin wall milling at a maximized axial depth of cut. In effect, it was possible to establish detailed relationships between tested variables.

The experimental design and results of the face-centred central composite design (FCCCD) are presented in Table A1.

## 3. Results

### 3.1. Cutting Force Components

**Figure 5:**
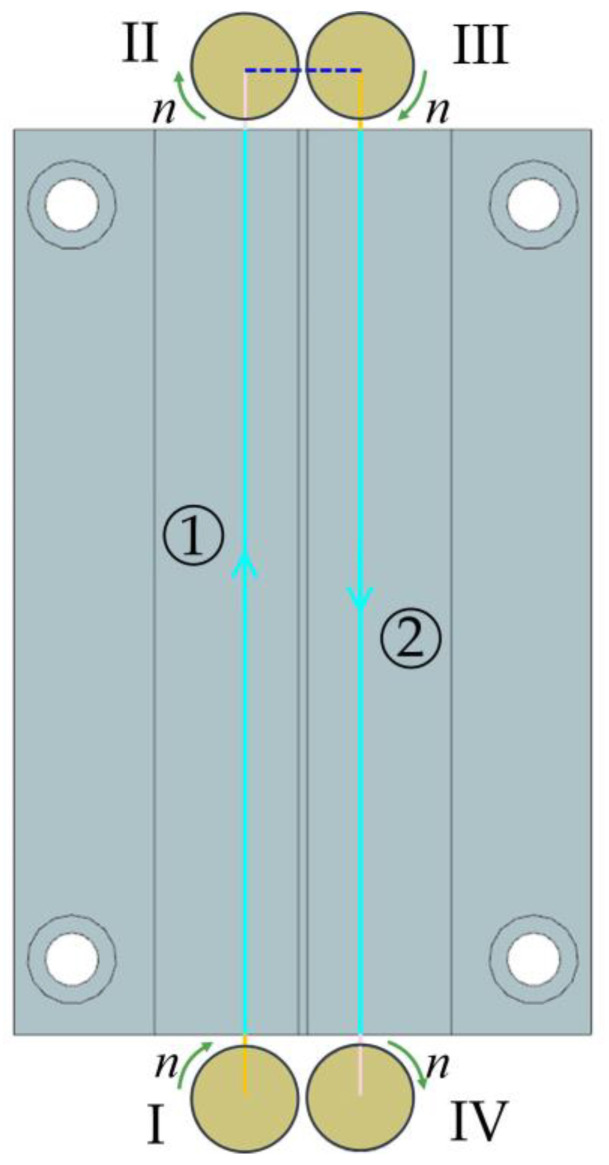
Cutting tool path during thin wall finishing at a maximized axial depth of cut: 1—first pass of the tool, 2—second pass of the tool, I and II—entry and exit of the tool during first pass, III and IV—entry and exit of the tool during second pass.

The next stage of the analysis of the results was the assessment of cutting force, which is an important criterion for describing the milling process. The cutting force reflects the real interaction conditions between the tool and the workpiece, and the examination of its behaviour pattern allows for the assessment of both material removal efficiency and machining system stability. For a more in-depth understanding of the cutting force components, Figure 5 shows a diagram illustrating the cutting tool path during thin wall finishing at a maximized axial depth of cut.

The machining operation was performed in two concurrent tool passes, denoted by numbers 1 and 2, respectively. Firstly, the tool made the first pass along the surface of the wall on the left side, in accordance with the assumed feed direction (marked with a blue arrow), starting from Point I, i.e., the tool entry into the material, until reaching Point II, marking the tool exit from the material after completing the first pass. After that, the tool made a second pass on the opposite side of the wall (on the right), in the reverse direction to the first pass (in accordance with the feed direction). Here, Point III marks the entry of the tool into the material during the second pass, while Point IV marks the moment when the tool exits the material after completing the machining. Green arrows indicate the direction of tool rotation.

**Figure 6:**
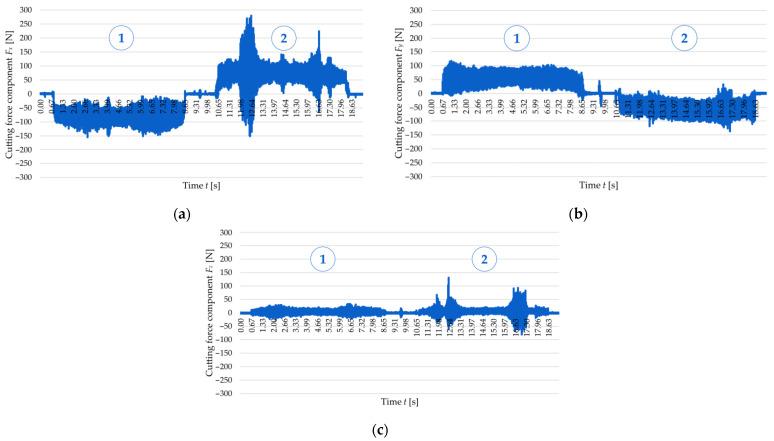
Variations in the cutting force components for a cutting speed of *v_c_* = 300 m/min and a wall thickness of *t* = 1 mm: (**a**) *F_x_*; (**b**) *F_y_*; (**c**) *F_z_*: 1—first tool pass, 2—second tool pass.

**Figure 7:**
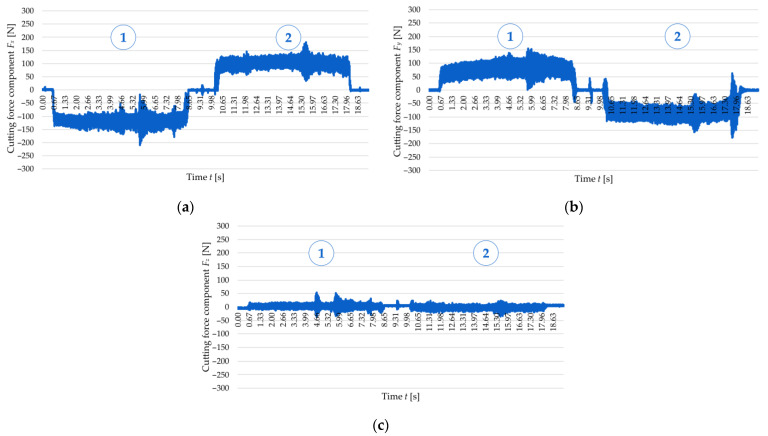
Variations in the cutting force components for a cutting speed of *v_c_* = 300 m/min and a wall thickness of *t* = 2 mm: (**a**) *F_x_*; (**b**) *F_y_*; (**c**) *F_z_*: 1—first pass of the tool, 2—second pass of the tool.

Figure 6 and Figure 7 show examples of time curves for individual cutting force components: *F_x_* (Figure 6a and Figure 7a), *F_y_* (Figure 6b and Figure 7b), and *F_z_* (Figure 6c and Figure 7c) for two tested wall thicknesses, *t* = 1 mm and *t* = 2 mm, for cutting speed *v_c_* = 300 m/min. The real-time recording of cutting force component signals allowed for observation of the cutting process dynamics and identification of load variations over time. An analysis of the curves indicates the occurrence of two cutting tool passes. The first pass takes place on the left side of the wall—following the orientation of the machine tool *y*-axis, while the second pass takes place on the right side of the wall, i.e., in the reverse direction to the *y*-axis of the machine tool. This method of performing the process results in a change in the direction of the cutting force, which was recorded in the form of a change in sign (from negative to positive or vice versa) of the *F_x_* and *F_y_* components. It should be emphasised that clear differences were observed in the curves obtained for these two cutting force components between the first and the second pass of the cutting tool. The first tool pass is characterised by greater stability, and there are no sudden jumps in the values of the cutting force components. In the second pass, however, the behaviour of the cutting force components becomes more unstable—there are numerous jumps in values and amplitude fluctuations, which suggests more difficult cutting conditions. The curves of the cutting force components *F_x_* and *F_y_*, especially of *F_x_*, show the presence of characteristic extremes, indicating temporary instabilities of the cutting process. This phenomenon can probably be linked to the occurrence of chatter, which results from the interaction between the cutting force and the dynamic susceptibility of the workpiece–tool–chuck–machine tool system. Additionally, when comparing the curves for the two analysed wall thicknesses (*t* = 1 mm and *t* = 2 mm), significant differences in the nature of the signal can be observed. For the thickness *t* = 1 mm, two distinct extremes are visible, while for *t* = 2 mm, only one such extreme was observed, which indicates a higher rigidity of this wall. The behaviour patterns of the components *F_x_* and *F_y_* at *t* = 1 mm are therefore characterised by greater instability and more dispersed signal fluctuations, while for a thickness of *t* = 2 mm, the variations are more orderly and regular. However, it is worth noting that the (absolute) maximum values of the cutting force components (without taking into account temporary extremes) were higher for the thickness *t* = 2 mm. The cutting force component *F_z_* has the least substantial effect on the cutting process due to the lowest values (in comparison to *F_x_* and *F_y_*). It should also be emphasised that the repeatability of the time curves for these cutting force components was confirmed via the central point runs according to FCCCD.

**Figure 8:**
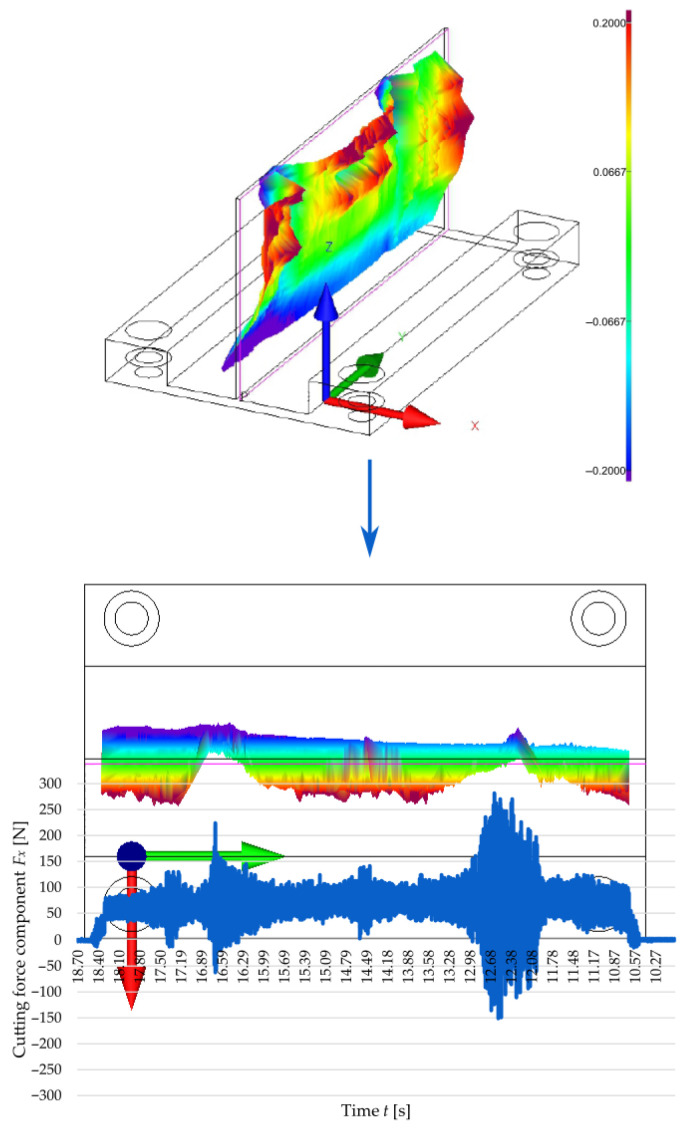
Correlation between the cutting force component *F_x_* during the second pass of the end mill at a cutting speed of *v_c_* = 300 m/min and a 3D visualisation of the flatness deviation in a thin wall with a thickness of *t* = 1 mm.

**Figure 9:**
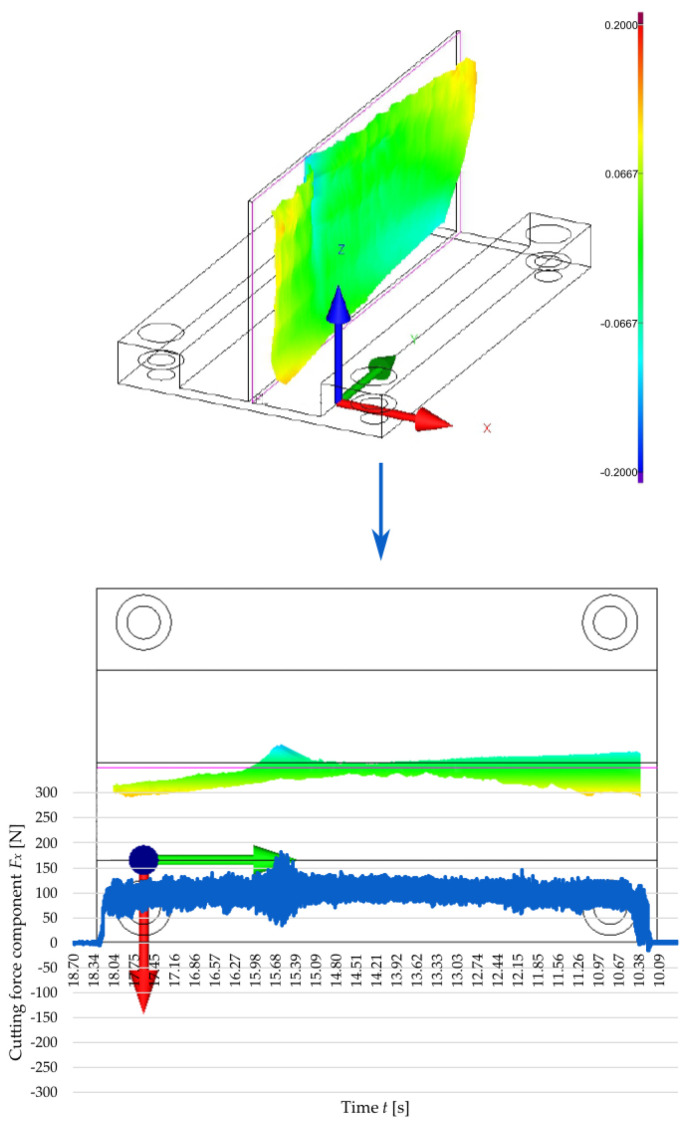
Correlation between the cutting force component *F_x_* during the second pass of the end mill at a cutting speed of *v_c_* = 300 m/min and a 3D visualisation of the flatness deviation in a thin wall with a thickness of *t* = 2 mm.

To establish a relationship linking the cutting force with the accuracy of manufactured thin walls, Figure 8 and Figure 9 show the correlation between the time curve of the *F_x_* observed for the second pass of the end mill at a cutting speed of *v_c_* = 300 m/min and the post-machining deformation of the thin walls with thicknesses *t* = 1 mm and *t* = 2 mm, respectively. The post-machining deformation was defined as a flatness deviation from the ideal reference plane (marked by pink outline). First, the focus was put on the wall with a thickness of *t* = 1 mm (Figure 8), and an analysis of the graphical representation of flatness deviation revealed a significant distortion of the wall. Next, the flatness deviation was shown in a top view, overlaid with a time curve of the *F_x_* for the second pass of the cutting tool, which was deliberately reversed to illustrate how the component varied depending on the milling path. In this way, it is possible to establish a correlation between two characteristic vertical marks (orthogonal to the tool feed direction) on the surface of a thin wall resulting from chatter, as well as the increased value and amplitude of the cutting force component *F_x_*. After that, a wall with a thickness of *t* = 2 mm was considered in the same way (Figure 9); for this case, the post-machining deformation of the wall was considerably smaller (compared to the wall thickness *t* = 1 mm). An analysis of the top view of the graphical representation of flatness deviation, combined with the time curve of the cutting force component *F_x_* demonstrates that the flatness deviation is significantly smaller and that the *F_x_* component curve is more stable. In addition, one characteristic vertical mark can be observed on the wall surface, which is related to the nature of variations in the cutting force component *F_x_*. In summary, the results obtained for the two tested wall thicknesses showed a clear correlation between the cutting force component *F_x_* and the post-machining deformation of the wall.

The next stage of the cutting force analysis was a quantitative assessment using mathematical modelling elements. The following part of this study offers a detailed analysis of *F_x_*, as this component had the most significant impact on the quality of thin walls. Its (absolute) maximum values were evaluated, omitting the characteristic extremes resulting from temporary instabilities of the machining process due to chatter. For a more accurate description of the process, a separate analysis was carried out for two passes of the cutting tool, denoting them by *F_x_*_1_ (first pass) and *F_x_*_2_ (second pass), respectively. This approach resulted from noticeable differences in the values of the cutting force component *F_x_* between these two passes. First, a statistical analysis of the experimental data for the first pass of the cutting tool was performed, and an empirical equation was determined to predict the value of the cutting force component *F_x_*_1_ as a function of two independent variables, i.e., cutting speed and wall thickness. This was performed via analysis of variance (ANOVA), which allows for the assessment of the significance of the impact of individual factors and the adjustment of a regression function. On this basis, it was found that the best fit to the experimental data was obtained for the quadratic model. The quality of the fit was assessed using the coefficients of determination:

- *R*^2^ = 0.9946;
- Adjusted *R*^2^ = 0.9907;
- Predicted *R*^2^ = 0.9675.

It should be emphasised that the difference between the predicted *R*^2^ and adjusted *R*^2^ values was less than 0.2, which indicates a good fit between the predictive model and the fitted model. In addition, the value of the determination coefficient *R*^2^ was close to 1. This indicates a very good fit of the empirical model to the experimental data. The high adequate precision value amounting to 44.2974 confirms the good signal-to-noise ratio, which is additional proof of the model’s reliability and its capacity to accurately replicate the relationship between independent and dependent variables. Ultimately, an empirical regression model was obtained describing the relationship between the cutting force component *F_x_*_1_ relative to the cutting speed and wall thickness, expressed via Equation (2) using coded variables:

(2) $$F_{x1}=282.64+22.11A+49.59B+8.12AB+0.6669A^{2}-89.92B^{2}$$

To verify the correctness and reliability of the developed model, a statistical verification was carried out using ANOVA for the adopted quadratic response surface model. This analysis allowed for the assessment of the statistical significance of the entire model and its individual components, as well as for determining the degree of fit of the model to the experimental data. The ANOVA results demonstrated the model’s *F*-value of 258.03, thus providing the model’s statistical significance. There was only a 0.01% probability that such a high *F*-value could occur by chance, i.e., as a result of measurement noise or random factors unrelated to the variables under study. This means that the wall thickness and the cutting speed have a significant impact on the shape of the response surface, and thus on the value of the *F_x_*_1_. A further analysis of the *p*-value confirmed that most model terms were statistically significant because they met the criterion *p* < 0.05. This means that the corresponding factors have a real impact on variations in the cutting force component *F_x_*_1_. It has been shown that both independent variables have a significant and strong impact on the *F_x_*_1_ during the first pass of the tool. The only exception is factor *A*^2^ (the square component of the variable describing wall thickness), which did not reach the statistical significance level. This indicates that the influence of wall thickness on *F_x_*_1_ is mainly linear and that non-linear relationships (of second order) are less significant in this case. The model fit quality was additionally verified by a lack-of-fit analysis. The obtained lack-of-fit *F*-value of 2.58 suggests that the lack of model fit is not statistically significant in relation to pure error. This means that the model accurately reproduces the actual measurement data, and any deviations are due to natural process variability rather than due to modelling errors. The probability that such a high lack of fit *F*-value could occur by chance is 19.09%, which confirms that there are no significant discrepancies between the model and the experimental data. It can therefore be stated that the developed quadratic model describing the cutting force component *F_x_*_1_ is statistically correct, well-fitted, and reliable. It can be effectively used to predict the value of the cutting force component *F_x_*_1_ during the first pass of the cutting tool under conditions similar to those applied in the experiments. Table 6 lists detailed results of ANOVA for the response surface quadratic model describing the cutting force component *F_x_*_1_ during the first pass of the cutting tool.

**Figure 10:**
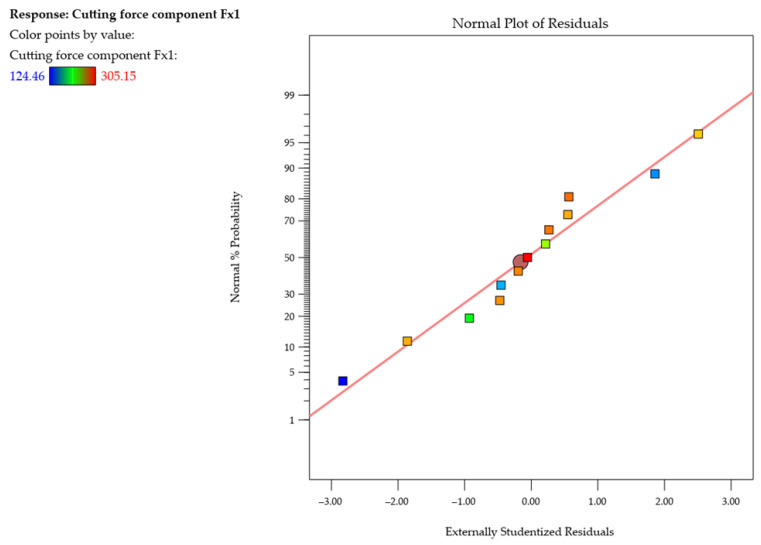
Normal probability plot of studentized residuals for the cutting force component *F_x_*_1_ (first pass of the cutting tool).

Since one of the key conditions for regression model accuracy is the assumption of normal distribution of residuals, Figure 10 presents the normal probability plot of studentized residuals for the cutting force component *F_x_*_1_ during the first pass of the cutting tool. An analysis of the plot shows that the points are distributed along a red straight line representing an ideal normal distribution. This arrangement indicates that the model residuals are symmetrically distributed around zero and that there are no significant deviations from the normal distribution. In particular, the lack of characteristic curvatures or clusters of points outside the reference line confirms that the model does not require transforming the response variable and that there are no outliers that could distort the analysis results. The normal probability plot of residuals thus confirms that the developed quadratic model of *F_x_*_1_ meets the assumption of the normality of residuals and can therefore be considered statistically correct and adequate for describing the cutting process under study.

**Figure 11:**
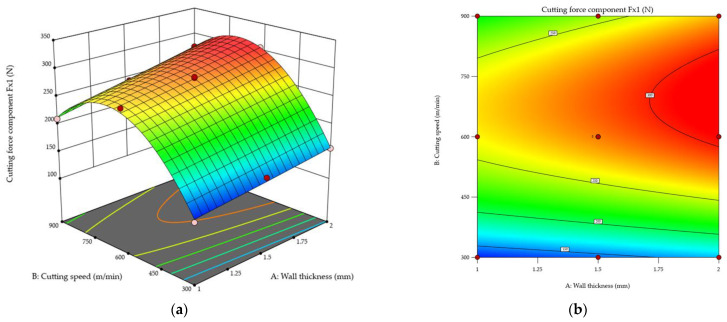
Cutting force component *F_x_*_1_ in the first pass of the cutting tool as a function of the wall thickness and cutting speed: (**a**) response surface plot; (**b**) contour plot.

Figure 11 shows the cutting force component *F_x_*_1_ during the first pass of the cutting tool as a function of the wall thickness and cutting speed. Results are given via response surface (Figure 11a) and contour (Figure 11b) plots. The results demonstrate a clear upward trend in the value of the cutting force component *F_x_*_1_ with increasing wall thickness. For the thinnest tested wall (*t* = 1 mm), the *F_x_*_1_ value is lowest, while a significant increase in this component value was observed with the use of a higher thickness. This probably results from the higher rigidity of this wall. The relationship between cutting speed and this force component is more complex. For each wall thickness, the values of the cutting force component *F_x_*_1_ increase with cutting speed until the maximum value is reached at a cutting speed of approximately *v_c_* ≈ 700 m/min, followed by a decrease in the value of *F_x_*_1_ with a further increase in the cutting speed. This is probably related to entering the high-speed cutting range. It is important to note that the transition from conventional machining to high-speed cutting differs depending on the material. However, it should be emphasised that the value of the cutting force component *F_x_*_1_ at *v_c_* = 900 m/min is still higher than at *v_c_* = 300 m/min. Furthermore, the shape of the resulting response surface is non-linear. The highest values of the cutting force component *F_x_*_1_ occurred for a combination of the greatest wall thickness *t* = 2 mm and a cutting speed of approximately *v_c_* ≈ 700 m/min, while the lowest values were obtained for the thinnest wall *t* = 1 mm and the lowest cutting speed *v_c_* = 300 m/min.

After that, the results of the cutting force component *F_x_*_2_ for the second pass of the end mill were analysed. Again, the (absolute) maximum values were compared, without taking into account the characteristic extremes resulting from chatter. First, a statistical analysis of the experimental data from the second pass of the cutting tool was performed, and an empirical equation was defined to predict the cutting force component *F_x_*_2_ as a function of the cutting speed and wall thickness. For this purpose, ANOVA was employed, and its results demonstrated that the quadratic model provided the best fit to the experimental data, yielding the following coefficients of determination:

- *R*^2^ = 0.9889;
- Adjusted *R*^2^ = 0.9810;
- Predicted *R*^2^ = 0.9553.

The high values of these coefficients (close to 1) confirm that the adopted quadratic model adequately describes the relationship between the independent variables and the cutting force component *F_x_*_2_. Furthermore, the difference between the predicted *R*^2^ and adjusted *R*^2^ values is less than 0.2. This indicates a good fit between the predictive model and the fitted model. The adequate precision value of 30.9410 confirms the good signal-to-noise ratio. Therefore, an empirical regression model was adopted to describe the cutting force component *F_x_*_2_ as a function of the cutting speed and wall thickness, expressed by Equation (3) with coded variables:

(3) $$F_{x2}=260.12+21.11A+47.25B+9.12AB+8.78A^{2}-91.81B^{2}$$

To determine the reliability of the adopted quadratic response surface model, a statistical verification was carried out. To that end, ANOVA was employed, enabling the assessment of the statistical significance of both the entire model and its individual components. In addition, this analysis also made it possible to determine the degree of fit of the theoretical model to the experimental data. As the model *F*-value is 124.94, the adopted model was found to be statistically significant. The probability that such a high *F*-value could occur by chance is only 0.01%. This means that the independent variables under study significantly affect the cutting force component *F_x_*_2_. An analysis of the *p*-values showed that most model terms met the significance criterion, i.e., *p* < 0.05, which indicates that they did affect the cutting force component *F_x_*_2_. It was found that the cutting speed had a strong influence on the cutting force component *F_x_*_2_, while the wall thickness is also an important factor, yet its influence is weaker. For the interactions *AB* and *A*^2^, no statistical significance was found. For *AB*, this indicates that wall thickness and cutting speed do not act synergistically and affect the cutting force component *F_x_*_2_ rather independently, while for *A*^2^, this shows that the effect of wall thickness on the cutting force component *F_x_*_2_ is primarily linear. For a more complete assessment of the model’s fit to the experimental data, a lack-of-fit analysis was also performed to determine whether the model deviates significantly from the actual data. The obtained lack-of-fit *F*-value of 0.7006 indicates that the lack of fit is not statistically significant, which means that the model accurately reflects the actual relationships between the variables. Concluding, the developed quadratic response surface model describing the cutting force component *F_x_*_2_ can be considered statistically reliable. This model can be effectively used to predict the cutting force component *F_x_*_2_ during the second pass of the cutting tool under similar experimental conditions. Table 7 lists ANOVA results for the response surface quadratic model describing the cutting force component *F_x2_* during the second pass of the cutting tool.

**Figure 12:**
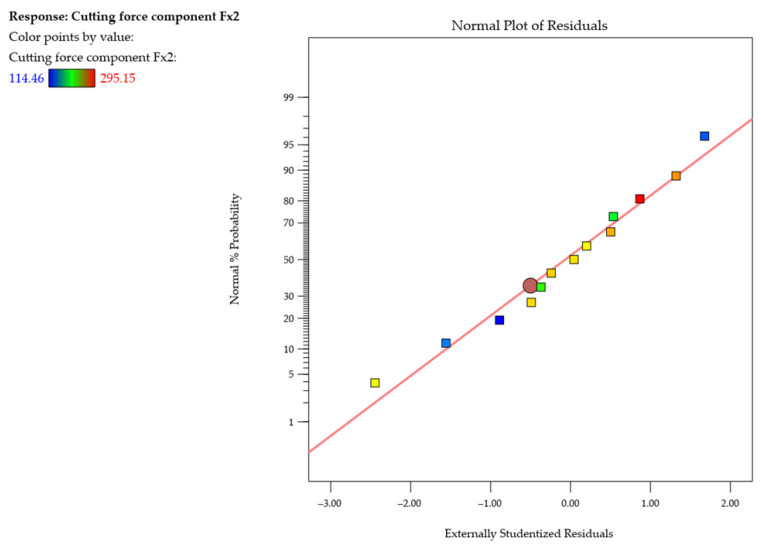
Normal probability plot of studentized residuals for the cutting force component *F_x2_* (second pass of the cutting tool).

Figure 12 shows the normal probability plot of studentized residuals for the cutting force component *F_x_*_2_ during the second pass of the end mill. On this basis, it was concluded that the residuals follow a straight line, suggesting that the model is adequate. No response transformation was observed, nor were there any significant deviations from the normal distribution (no curvatures, no point clusters, no outliers).

**Figure 13:**
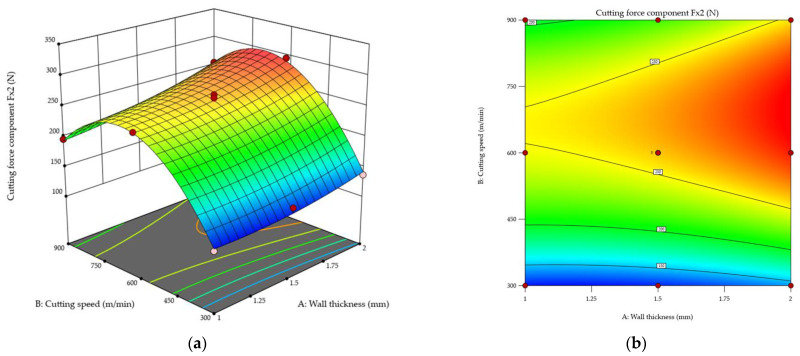
Cutting force component *F_x_*_2_ during the second pass of the cutting tool as a function of the wall thickness and cutting speed: (**a**) response surface plot; (**b**) contour plot.

Relationships between the cutting force component *F_x_*_2_ during the second pass of the cutting tool and the wall thickness and cutting speed are shown in Figure 13 as response surface (Figure 13a) and contour (Figure 13b) plots, demonstrating that the values of the cutting force component *F_x_*_2_ increase with wall thickness. The lowest values of the cutting force component *F_x_*_2_ were observed for a wall thickness of *t* = 1 mm, while the highest values were obtained for *t* = 2 mm. The same correlation between cutting speed and the cutting force component *F_x_*_2_ was observed for each tested wall thickness. A cutting speed increase causes an increase in the cutting force component *F_x_*_2_ until it reaches the maximum value at a cutting speed of approximately *v_c_* ≈ 700 m/min, but a further increase in the cutting speed causes the cutting force component *F_x_*_2_ to decrease. Importantly, the values of the cutting force component *F_x_*_2_ at *v_c_* = 900 m/min are still higher than those observed at *v_c_* = 300 m/min. Concluding, the highest values of the cutting force component *F_x_*_2_ were obtained for a combination of the thickest wall *t* = 2 mm and a cutting speed of approximately *v_c_* ≈ 700 m/min, while the lowest values were obtained for the thinnest wall *t* = 1 mm and the lowest cutting speed *v_c_* = 300 m/min. Furthermore, it should be emphasised that differences were found between the maximum values of the cutting force components *F_x_*_1_ and *F_x_*_2_ in two passes of the cutting tool. During the second pass of the end mill, the cutting force component *F_x_*_2_ was lower (in relation to *F_x_*_1_).

The results demonstrate that both independent variables affect the cutting force, specifically the components of this force.

### 3.2. Cutting Temperature

The final stage of the analysis of the obtained test results involved measuring the cutting temperature *T* by the natural thermocouple method. Focus was put on the maximum values of electromotive force resulting from a potential difference between two elements of this thermocouple: the workpiece and the cutting tool. Based on a developed reference curve and an equation describing this curve, the corresponding temperatures were determined. This made it possible to convert the electromotive force values into the actual temperature in the contact zone between the cutting tool and the workpiece. Regarding the cutting temperature *T*, no differences were observed between the first and second passes of the cutting tool; therefore, the maximum temperature values obtained during the machining of both sides of the wall were averaged. It should be noted that the cutting temperature was measured directly at the interface between the cutting edge and the workpiece. Due to the tool geometry and process kinematics, this contact zone can be considered point-like. The cutting temperature was the average of ten maximum peaks observed in the analysed end mill passes. After that, a statistical analysis of the experimental data was performed, and an empirical equation was determined to predict the cutting temperature *T* as a function of the cutting speed and wall thickness. The ANOVA was used for this purpose, and the results demonstrated the best fit of the quadratic model to the experimental data, which is confirmed by the following coefficients:

- *R*^2^ = 0.9911;
- Adjusted *R*^2^ = 0.9847;
- Predicted *R*^2^ = 0.9812.

The values of the coefficients of determination are close to 1, which confirms that the quadratic model accurately describes the relationship between the cutting speed and wall thickness and the cutting temperature *T*. Additionally, the difference between the predicted *R*^2^ and adjusted *R*^2^ values does not exceed 0.2, which indicates a good fit between the predictive model and the fitted model. The adequate precision value of 31.5254 confirms a favourable signal-to-noise ratio, which means that the model is characterised by high prediction quality and stability in terms of the analysed data. On this basis, an empirical regression model was adopted to describe the cutting temperature *T* as a function of the cutting speed and wall thickness, expressed by Equation (4) using coded variables:

(4) $$T=355.55+1.50A+17.50B-0.2500AB+3.57A^{2}-38.43B^{2}$$

To assess the reliability of the adopted quadratic response surface model, it was verified statistically by ANOVA. The obtained model *F*-value of 155.98 indicates the model’s statistical significance. The probability of obtaining such a high *F*-value by chance was estimated at 0.01%, which clearly confirms that the independent variables affect the cutting temperature *T*. An analysis of the significance levels (*p*-value) showed that almost half of the components included in the model met the significance criterion (*p* < 0.05). This means that they have a real impact on variations in the cutting temperature *T*. Among the factors under consideration, cutting speed has the greatest impact, while wall thickness proved to be a statistically insignificant variable. The interaction components *AB* and *A*^2^ were found to bear no statistical significance either. Additionally, a lack-of-fit analysis was performed. The obtained lack-of-fit *F*-value of 0.1571 does not indicate significant differences between the predicted and experimental values. This proves that the model accurately reproduces the actual relationships between the analysed process variables. The developed quadratic response surface model can therefore be considered statistically correct and reliable. It is characterised by a good fit to the experimental data and high predictive power, and can thus be effectively used to predict the cutting temperature *T* under similar experimental conditions. Obtained ANOVA results for the response surface quadratic model describing the cutting temperature *T* are listed in Table 8.

**Figure 14:**
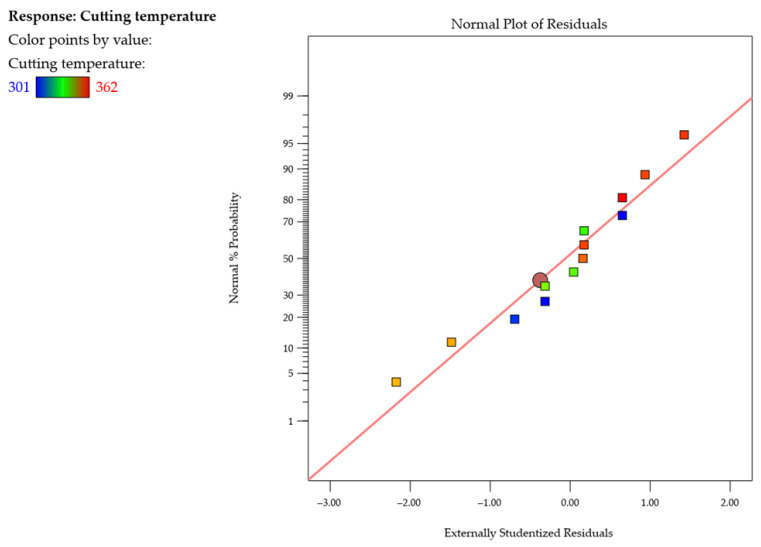
Normal probability plot of studentized residuals for the cutting temperature *T*.

An analysis of the normal probability plot of the studentized residuals for the cutting temperature *T* shown in Figure 14 reveals that the residuals follow a straight line, which suggests that the model is adequate. In addition to that, the plots show no response transformations or normal distribution problems.

**Figure 15:**
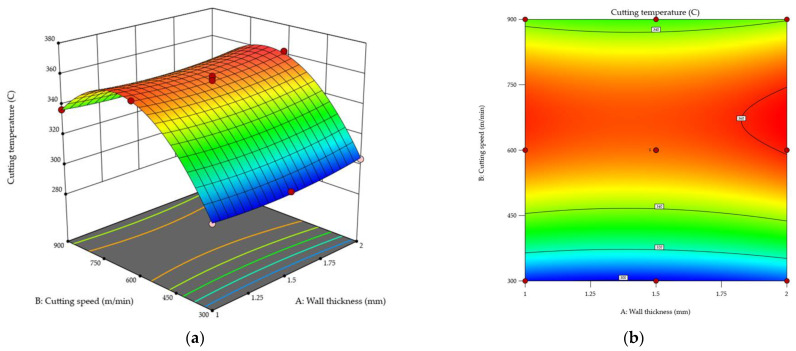
Cutting temperature *T* as a function of wall thickness and cutting speed: (**a**) response surface plot; (**b**) contour plot.

Figure 15 shows the pattern of variations in the cutting temperature T as a function of the cutting speed and wall thickness. The analysis was based on response surface (Figure 15a) and contour (Figure 15b) plots. A merely slight increase in the temperature *T* was observed with increasing the wall thickness, and the ANOVA results confirmed a lack of statistical significance for this variable. It was found that, whatever the wall thickness, the cutting temperature *T* would increase with increasing the cutting speed up to *v_c_* ≈ 700 m/min, while a further raising to *v_c_* = 900 m/min would cause a slight decrease in the temperature. The maximum cutting temperature *T* of about 360 °C was observed for a wall thickness of *t* = 2 mm and a cutting speed of approximately *v_c_* ≈ 700 m/min, while the lowest value of *T* was obtained for the thinnest wall *t* = 1 mm and the lowest cutting speed *v_c_* = 300 m/min.

The results demonstrate that the cutting temperature *T* primarily depends on the cutting speed.

## 4. Discussion

An analysis of the results confirms the key role of thermomechanical interactions in thin wall milling at a maximum axial depth of cut, where thermal and mechanical effects occur simultaneously and are intertwined. The cutting force in this process was found to be significantly affected by both wall thickness and cutting speed, while the cutting temperature was primarily dependent on the cutting speed.

The results of the cutting force components showed that the *F_x_* plays a key role in the identification of mechanical interactions in thin wall milling at a maximized axial depth of cut. The maximum values of the *F_x_* component were lower in the second pass of the tool than in the first pass. Also, during the machining of the other side of the wall, there were significant fluctuations in the amplitude of this component, indicating the instability of the machining process. The fluctuations probably resulted from a reduced rigidity [59] of the wall in the second tool pass (due to the removed machining allowance in the first pass). This phenomenon was particularly pronounced for the thinnest tested wall (*t* = 1 mm), while the highest values of the cutting force component *F_x_* (excluding temporary extremes) were observed for the thickest tested wall *t* = 2 mm. This was likely related to the fact that as the wall thickness was increased, its susceptibility to elastic-plastic deformation under the cutting force decreased (for analysed wall thicknesses); hence, it can be concluded that the thicker the wall, the higher the observed cutting force value. This effect can be attributed to the low stiffness of thin-walled elements [36]. The cutting speed was found to have a non-linear impact on the cutting force component *F_x_*. An increase in the value of this component was observed when the cutting speed was increased to approximately ≈ 700 m/min, while a further increase in *v_c_* led to its decrease. This phenomenon can be linked to entering the high-speed cutting range, which results from a decrease in the friction factor and a lower share of strains in the total energy balance of the process, leading to a reduced mechanical load [30,60]. This is due to a shorter heat-exposure time of the tool and the workpiece, predominance of chip heat dissipation, and thermomechanical effects such as adiabatic chip shear [61]. In addition to that, a close correlation was observed between the cutting force component *F_x_* and the post-machining deformation of a thin wall [62]. The areas with the highest amplitude of this component corresponded to the locations of local distortions and characteristic vertical marks on the surface. Two such marks were observed for a wall thickness of *t* = 1 mm and one for a thickness of *t* = 2 mm (for *v_c_* = 300 m/min), which confirms that the process stability increased with increasing wall thickness.

The cutting temperature *T* was found to depend mainly on the cutting speed [63]. An increase in the temperature was observed as the cutting speed was increased to *v_c_* ≈ 700 m/min, while a further raising the cutting speed to *v_c_* = 900 m/min resulted in a slight decrease in the temperature value. The maximum temperature *T* of about 360 °C was observed for a wall thickness of *t* = 2 mm and a cutting speed of approximately *v_c_* ≈ 700 m/min, while the lowest temperature was measured for the thinnest tested wall, *t* = 1 mm, and the lowest cutting speed *v_c_* = 300 m/min. The temperature variations were primarily due to increasing friction between the tool, the chip, and the workpiece as the cutting speed was increased [64]. The temperature decrease observed in the range of *v_c_* = 700–900 m/min probably resulted from lower cutting resistance, a phenomenon which is characteristic of high-speed cutting [65].

The empirical models developed using the response surface method showed a very good fit to the experimental data (the coefficient of determination *R*^2^ > 0.98), which confirms their high predictive reliability. The ANOVA results showed both the wall thickness and cutting speed to be statistically significant for the cutting force component *F_x_*, while for the cutting temperature *T*, the only statistically significant variable was cutting speed.

Summing up, the results have demonstrated that walls with thicknesses of *t* ≥ 1.5 can be milled at a maximum axial depth of cut, provided that the cutting speed is selected appropriately and the machining process stability is ensured. The examination of thermomechanical interactions has confirmed their key role in the post-machining deformation of thin walls made of the 7050 T7451 alloy. Thermal and mechanical phenomena occur simultaneously and interact with each other, thus affecting the stability and accuracy of the machining process. Additionally, it should be emphasised that this study is a continuation of the author’s research presented in the work [53]. A key issue when milling thin-walled components is the prevention of chatter [22,66] by optimisation of process parameters [67]. Therefore, further studies should include a modal analysis of the entire system (tool–holder–spindle–workpiece), which allows for the development of stability lobe diagrams showing the stability limits of a milling process as a function of the spindle speed and axial depth of cut (less often, radial depth of cut, i.e., milling width) [68,69,70].

## 5. Conclusions

The results of this study lead to the following conclusions:

- The study showed that the highest maximum values (excluding characteristic extremes resulting from temporary process instability due to chatter) were obtained for the cutting force component *F_x_* compared to the other two components, *F_y_* and *F_z_*. The *F_x_* component was found to have a decisive impact on the post-machining deformation of thin walls. An analysis of the time curves showed that an increase in the amplitude of this component resulted in wall distortion and the formation of characteristic vertical marks on the machined surface.
- The highest values of *F_x_* were observed at a cutting speed of approximately ≈ 700 m/min, while a further increase in the speed led to a decrease in this force component. This phenomenon can be associated with the transition from conventional machining to high-speed cutting, where friction and plastic stress in the cutting zone are reduced, leading to lower cutting resistance. Despite a partial decrease in *F_x_* at the highest tested speed of *v_c_* = 900 m/min, its value remained significantly higher than that observed at *v_c_* = 300 m/min.
- For the thinnest tested wall, *t* = 1 mm, the greatest instability of the machining process and clear signs of chatter were observed, while an increase in the wall thickness to *t* = 2 mm resulted in enhanced process stability and reduced post-machining deformation. This was due to an increase in the rigidity of the workpiece, which reduced its susceptibility to chatter. At the same time, the results confirmed that in terms of achieving the required dimensional and shape accuracy, the wall thickness value *t* ≥ 1.5 mm was the minimum for milling at a maximized axial depth of cut.
- Regarding the impact of wall thickness on the cutting force component *F_x_*, the results showed that an increase in the wall thickness value would lead to a higher *F_x_* component (for analysed wall thicknesses). The observed behaviour is probably related to the fact that, as the wall thickness was increased, the stiffness of the workpiece increased too, thereby reducing its susceptibility to elastic-plastic deformation under the cutting force. As a result, the cutting force values vary, since the formation of wall deflection during machining alters the cross-section of the material being removed.
- The cutting temperature *T* primarily depends on the cutting speed. It was observed that the temperature increased as the cutting speed was increased to *v_c_* ≈ 700 m/min, whereas a further increase in the cutting speed to *v_c_* = 900 m/min led to a slight decrease in the temperature. The maximum observed temperature *T* was approximately 360 °C.
- The empirical models developed using response surface methodology showed a very high level of fit (*R*^2^ > 0.98). This proves their suitability for predicting the tested variables under similar machining conditions. The ANOVA results demonstrated the statistical significance of both cutting force and wall thickness for the cutting force component *F_x_*, while for the cutting temperature *T,* it was only cutting speed that was statistically significant.
- The dominant factors inducing the post-machining deformation of thin walls include the interaction between cutting force and heat generated in the cutting zone, as well as the occurrence of chatter. These mechanical and thermal effects are closely related, and their interaction affects the quality of a finished product.
- Thin wall milling at a maximized axial depth of cut is possible but requires ensuring adequate dynamic stability of the tool–holder–spindle system, as well as taking into account the aspect of workpiece rigidity, which is a complex issue in the case of thin-walled structural components. In the future, the modal analysis for the whole system should be performed.

The proposed integrated research approach related to the thermomechanical interaction analysis, including cutting force and cutting temperature, is an effective tool for optimising the machining parameters of thin-walled components, ensuring their dimensional and shape accuracy. The combination of these solutions makes it possible not only to predict process behaviour under industrial conditions, but also to consciously control technological parameters in order to minimise deformation and improve the quality of manufactured components.

## Figures and Tables

**Table 1:** Comparison of initial residual stress and machining-induced residual stress.

Characteristic	Initial Residual Stress	Machining-Induced Residual Stress	Reference
Formation mechanism	Manufacturing processes for semi-finished products (rolling, casting, forging, thermal treatment, etc.)	Cutting processes	[43]
Processes	Plastic deformation, distortion by rolling or forging, thermal gradient by heat treatment	Plastic deformation, cutting force, cutting temperature, phase transformation	[44]
Depth of location and distribution	Over the entire material thickness, symmetrically distributed relative to the centre of the material	Usually, at depths up to 1 mm, the maximum stress is located at a certain distance from the surface	[45]
Effect on the distortion of thin-walled components	Greatest impact on the distortion of a workpiece during roughing	Greatest impact on the distortion of a workpiece during finishing (of key significance for wall thicknesses below 4–5 mm)	[46]
	Main cause of the distortion of thick parts	Main source of the distortion of thin-walled parts	[44]

**Table 2:** Independent variables and their coded levels for the face-centred central composite design (FCCCD).

Independent Variables	Codes	Levels of Coded Variables		
		Low	Medium	High
		−1	0	+1
Wall thickness *t* [mm]	*A*	1	1.5	2
Cutting speed *v_c_* [m/min]	*B*	300	600	900

**Table 3:** Chemical composition and selected mechanical properties of the 7050 T7451 alloy (compiled based on [58]).

Chemical Composition [%]											
Si	Fe	Cu	Mn	Mg	Cr	Zn	Ti	Zr	Others	Others Total	Al
0.05	0.07	2.20	0.01	2.10	0.01	6.30	0.03	0.10	0.01	0.03	The rest
**Mechanical Properties**											
**Tensile Strength** ***R_m_* [MPa]**				**Yield Strength** ***R_p_*_0.2_ [MPa]**				**Elongation** ***A*_5_ [%]**			
520				450				13			

**Table 4:** Technological parameters of roughing and finishing operations.

Cutting Parameters	Roughing	Finishing
Cutting speed *v_c_* [m/min]	900	300, 600, 900
Feed per tooth *f_z_* [mm/tooth]	0.05	0.025
Axial depth of cut *a_p_* [mm]	5 and 3 (for the final tool pass)	48
Radial depth of cut (milling width) *a_e_* [mm]	8.9 and 10.4 (for a wall thickness of 1 mm)	0.2
	8.65 and 10.4 (for a wall thickness of 1.5 mm)	
	8.4 and 10.4 (for a wall thickness of 2 mm)	

**Table 5:** Detailed technical parameters of cutting tools used for roughing and finishing.

Technical Parameters	44985	44748
Cutting diameter [mm]	16	12
Shank diameter [mm]	16	12
Length of cut [mm]	35	48
Overall length [mm]	108	100
Helix angle [°]	Variable	41
Number of flutes [-]	3	4
Coating	TiB_2_	TiB_2_

**Table 6:** ANOVA for the response surface quadratic model of a cutting force component *F_x_*_1_ (first pass of the cutting tool).

Source	Sum of Squares	df	Mean Square	*F*-Value	*p*-Value	
**Model**	43,927.34	5	8785.47	258.03	<0.0001	significant
*A*-Wall thickness	2932.23	1	2932.23	86.12	<0.0001	
*B*-Cutting speed	14,753.03	1	14,753.03	433.29	<0.0001	
*AB*	263.74	1	263.74	7.75	0.0272	
*A* ^2^	1.23	1	1.23	0.0361	0.8547	
*B* ^2^	22,333.22	1	22,333.22	655.92	<0.0001	
**Residual**	238.34	7	34.05			
Lack of Fit	157.17	3	52.39	2.58	0.1909	not significant
Pure Error	81.17	4	20.29			
**Cor Total**	44,165.68	12				

Standard deviation: 5.84, mean: 241.44, coefficient of variation %: 2.42, *R*^2^: 0.9946, adjusted *R*^2^: 0.9907, predicted *R*^2^: 0.9675, adequate precision: 44.2974.

**Table 7:** ANOVA for the response surface quadratic model of the cutting force component *F_x2_* (second pass of the cutting tool).

Source	Sum of Squares	df	Mean Square	*F*-Value	*p*-Value	
**Model**	41,902.95	5	8380.59	124.94	<0.0001	significant
*A*-Wall thickness	2672.95	1	2672.95	39.85	0.0004	
*B*-Cutting speed	13,397.27	1	13,397.27	199.72	<0.0001	
*AB*	332.70	1	332.70	4.96	0.0612	
*A* ^2^	212.73	1	212.73	3.17	0.1182	
*B* ^2^	23,282.23	1	23,282.23	347.08	<0.0001	
**Residual**	469.56	7	67.08			
Lack of Fit	161.74	3	53.91	0.7006	0.5991	not significant
Pure Error	307.82	4	76.96			
**Cor Total**	42,372.51	12				

Standard deviation: 8.19, mean: 221.79, coefficient of variation %: 3.69, *R*^2^: 0.9889, adjusted *R*^2^: 0.9553, predicted *R*^2^: 0.9553, adequate precision: 30.9410.

**Table 8:** ANOVA for the response surface quadratic model for the cutting temperature *T*.

Source	Sum of Squares	df	Mean Square	*F*-Value	*p*-Value	
**Model**	6326.45	5	1265.29	155.98	<0.0001	significant
*A*-Wall thickness	13.50	1	13.50	1.66	0.2380	
*B*-Cutting speed	1837.50	1	1837.50	226.51	<0.0001	
*AB*	0.2500	1	0.2500	0.0308	0.8656	
*A* ^2^	35.18	1	35.18	4.34	0.0758	
*B* ^2^	4079.18	1	4079.18	502.85	<0.0001	
**Residual**	56.78	7	8.11			
Lack of Fit	5.98	3	1.99	0.1571	0.9199	not significant
Pure Error	50.80	4	12.70			
**Cor Total**	6383.23	12				

Standard deviation: 2.85, mean: 339.46, coefficient of variation %: 0.8390, *R*^2^: 0.9911, adjusted *R*^2^: 0.9847, predicted *R*^2^: 0.9812, adequate precision: 31.5254.

## Data Availability

The original contributions presented in this study are included in the article. Further inquiries can be directed to the corresponding author.

## Appendix A

**Table A1:** Experimental design and results of face-centred central composite design (FCCCD).

Run	Actual Variable 1: Wall Thickness *A* [mm]	Coded Variable 1: Wall Thickness *A* [-]	Actual Variable 2: Cutting Speed *B* [m/min]	Coded Variable 2: Cutting Speed *B* [-]	Response 1: Cutting Force Component *F_x_*_1_ [N]	Response 2: Cutting Force Component *F_x_*_2_ [N]	Response 3: Cutting Temperature *T* [°C]
1	1	−1	300	−1	124.46	114.46	301
2	1.5	0	300	−1	149.76	129.76	301
3	1.5	0	600	0	281.52	269.48	358
4	1	−1	900	+1	210.23	196.23	337
5	2	+1	600	0	305.15	295.15	362
6	1.5	0	600	0	279.98	256.25	359
7	1.5	0	900	+1	243.26	213.26	335
8	1	−1	600	0	269.05	249.05	358
9	2	+1	300	−1	156.49	136.49	304
10	1.5	0	600	0	274.14	264.08	356
11	2	+1	900	+1	274.74	254.74	339
12	1.5	0	600	0	284.15	258.19	352
13	1.5	0	600	0	285.79	246.19	351
